# Supplementary material for: Identification and Molecular Characterization of a Divergent Asian-like Canine Parvovirus Type 2b (CPV-2b) Strain in Southern Italy
Source: Int J Mol Sci. 2022 Sep 24;23(19):11240. doi: 10.3390/ijms231911240 (PMC9570342; doi:10.3390/ijms231911240)
Supplement: Supplementary file 1 [file ijms-23-11240-s001.zip › ijms-1897345-supplementary.pdf]

Supplementary Material Figure S1. Close relatives to the CPV-2b strain identified in this study.

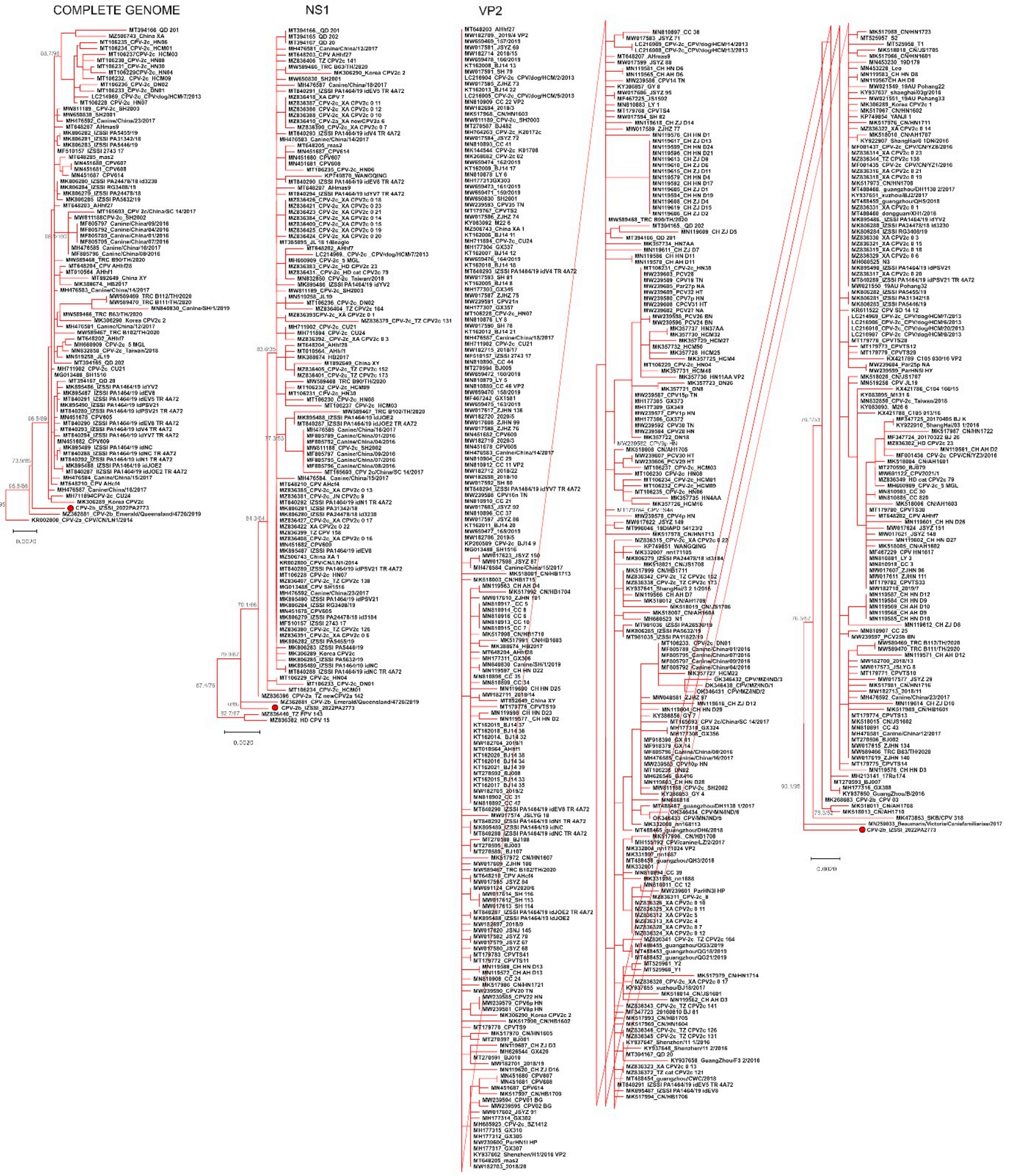

Supplementary Material Figure S2. Sequence analysis of Asian CPV-2c-like strains key amino acid residues.

Asian CPV-2-like

NS1

N	Residue 60	544	545	630
110	V	F	V	P
2	I	F	V	P
1	V	Y	E	P

VP2

Type	N	Residue 5	267	297	324	370	426	440
c	368	G	Y	A	I	R	E	T
	111	A	Y	A	I	R	E	T
	6	G	Y	A	I	Q	E	T
	4	A	Y	A	I	Q	E	T
	1	G	Y	A	I	R	E	A
	1	G	Y	A	L	R	E	T
b	1	G	Y	A	I	R	D	T
	1	G	Y	A	I	Q	D	T
	1	A	Y	A	I	R	D	T
a	1	G	F	A	I	Q	N	T
	1	G	Y	A	I	R	N	A
	1	A	Y	A	I	R	N	T
	1	G	Y	A	I	Q	N	T

Other clades

NS1

N	Residue 60	544	545	630
355	I	Y	E	L
96	I	F	E	L
35	I	Y	Q	L
34	I	F	V	L
7	V	Y	E	L
2	I	Y	K	L
1	V	F	E	L
1	I	F	Q	L
1	I	Y	V	L

VP2

Type	N	Residue 5	267	297	324	370	426	440
c	417	A	F	A	Y	Q	E	T
	50	A	F	A	Y	Q	E	A
	9	A	Y	A	I	R	E	T
	3	A	Y	A	I	Q	E	T
	2	A	F	N	N	Q	E	T
	2	A	F	A	Y	Q	E	S
	1	A	F	S	Y	Q	E	T
	1	G	Y	A	I	Q	E	T
	1	A	Y	A	I	Q	E	A
	1	A	F	A	Y	R	E	T
	1	A	Y	A	Y	Q	E	A
	1	A	Y	A	Y	Q	E	T
	1	F	F	A	Y	Q	E	T
b	392	A	F	A	Y	Q	D	T
	154	A	Y	A	I	Q	D	A
	48	A	Y	A	I	Q	D	T
	44	G	Y	A	Y	Q	D	T
	23	A	Y	A	Y	Q	D	T
	21	A	F	S	Y	Q	D	T
	12	A	F	N	Y	Q	D	T
	6	A	F	N	L	Q	D	T
	5	A	F	A	L	Q	D	T
	3	A	F	A	Y	Q	D	S
	2	A	F	A	I	Q	D	T
	2	A	F	N	I	Q	D	T
	2	A	F	A	Y	Q	D	A
	1	A	L	A	Y	Q	D	T
	1	A	F	S	Y	Q	D	A
	1	G	Y	A	I	Q	D	A
	1	A	Y	T	I	Q	D	T
	1	A	Y	A	N	Q	D	T
	1	A	Y	A	Y	Q	D	A
?	7	A	F	S	Y	Q	K	T
	5	T	F	S	Y	Q	K	T

Type	N	Residue 5	267	297	324	370	426	440
a/FPV	846	A	F	S	Y	Q	N	T
	591	A	Y	A	I	Q	N	A
	398	A	F	A	Y	Q	N	T
	229	A	F	A	I	Q	N	T
	51	A	F	A	Y	Q	N	A
	21	A	Y	A	I	Q	N	T
	18	A	F	A	L	Q	N	T
	18	A	F	S	Y	Q	N	T
	17	A	F	A	Y	Q	N	S
	11	G	Y	A	Y	Q	N	T
	7	A	F	A	I	Q	N	A
	6	G	Y	A	I	Q	N	A
	6	G	F	A	I	Q	N	T
	6	A	F	A	I	R	N	T
	6	A	F	N	Y	Q	N	T
	4	G	F	A	Y	Q	N	T
	4	A	F	N	Y	Q	N	A
	3	A	Y	A	Y	Q	N	T
	2	A	F	S	Y	Q	N	R
	2	G	F	S	Y	Q	N	T
	1	G	Y	S	Y	Q	N	T
	1	P	Y	A	I	Q	N	A
	1	A	Y	A	T	Q	N	A
	1	A	Y	A	I	K	N	A
	1	V	F	A	I	Q	N	T
	1	A	F	A	L	Q	N	A
	1	A	F	S	I	Q	N	A
	1	A	F	S	I	Q	N	T
	1	A	F	A	N	Q	N	T
	1	A	F	S	C	Q	N	T
	1	A	F	N	I	Q	N	T
	1	A	F	S	H	Q	N	T
	1	A	F	S	Y	Q	N	S
	1	P	F	S	Y	Q	N	T
	1	A	F	A	F	Q	N	A

Supplementary Material Table S1. Complete genomes, NS1 and VP2 CPV-2 strains showing the highest nucleotide identities to the identified strain.

% Id.	CPV-2 type	Strain/Isolate	Acc.nr.	Host	Country	Year
		Nearly complete genome				
99.60	CPV-2c	CPV609	MN451682	Dog	Nigeria	2018
99.58	CPV-2c	CPV-SH1516	MG013488	Dog	Cina	2017
	CPV-2c	CPV605	MN451678	Dog	Nigeria	2018
	CPV-2c	IZSSI_PA1464/19_idNC	MK895489	Dog	Nigeria	2018
	CPV-2c	IZSSI_PA1464/19_idPSV21	MK895490	Dog	Nigeria	2018
	CPV-2c	IZSSI_PA1464/19_idN1_TR_4A72	MT840292	Dog	Nigeria	2018
	CPV-2c	IZSSI_PA1464/19_idV4_TR_4A72	MT840293	Dog	Nigeria	2018
99.55	CPV-2c	CU21	MH711902	Cat	Thailand	2016
	CPV-2c	Canine/China/18/2017	MH476587	Dog	China	2017
	CPV-2c	CPV_IZSSI_2743_17	MF510157	Dog	Italy	2017
	CPV-2c	IZSSI_PA1464/19_idEV8	MK895487	Dog	Nigeria	2018
	CPV-2c	IZSSI_PA1464/19_idEV5_TR_4A72	MT840291	Dog	Nigeria	2018
	CPV-2c	IZSSI_PA1464/19_idYV7_TR_4A72	MT840294	Dog	Nigeria	2018
		NS1 gene				
99.75	CPV-2a	CPV/CN/LN1/2014	KR002800	Dog	China	2014
	CPV-2c	CPV-SH1516	MG013488	Dog	China	2017
	CPV-2c	Canine/China/23/2017	MH476592	Dog	China	2017
	CPV-2c	Canine/China/14/2017	MH476583	Dog	China	2017
	CPV-2c	XA-CPV2c-0-17	MZ836427	Dog	China	2019
	CPV-2c	XA-CPV2c-0-22	MZ836422	Dog	China	2019
	CPV-2c	XA-CPV2c-0-16	MZ836408	Dog	China	2019
	CPV-2c	TZ-CPV2c-138	MZ836407	Dog	China	2019
	n.a.	TZ-CPV-158	MZ836399	Dog	China	2019
	CPV-2a	TZ-newCPV2a-142	MZ836396	Dog	China	2019
	CPV-2c	XA-CPV2c-0-6	MZ836391	Dog	China	2019
	CPV-2c	XA-CPV2c-0-13	MZ836385	Dog	China	2019
	CPV-2c	JN-CPV2c-9	MZ836381	Dog	China	2019
	CPV-2c	TZ-CPV2c-126	MZ836380	Dog	China	2019
	CPV-2c	CPV-AHcf4	MT648210	Dog	China	2019
	CPV-2c	China-XA-1	MZ506743	Dog	China	2019
	CPV-2c	HN07	MT106228	Dog	Vietnam	2017
	CPV-2c	CPV_IZSSI_2743_17	MF510157	Dog	Italy	2017
	CPV-2c	IZSSI_PA24478/18_id3184	MK806279	Dog	Italy	2018
	CPV-2c	IZSSI_PA24478/18_id3230	MK806280	Dog	Italy	2018
	CPV-2c	IZSSI_PA31342/18	MK806281	Dog	Italy	2018
	CPV-2c	CPV609	MN451682	Dog	Nigeria	2018
	CPV-2c	CPV605	MN451678	Dog	Nigeria	2018
	CPV-2c	IZSSI_PA1464/19_idNC	MK895489	Dog	Nigeria	2018
	CPV-2c	IZSSI_PA1464/19_idPSV21	MK895490	Dog	Nigeria	2018
		VP2 gene				
	CPV-2c	CPV-VT12	KP715688	Dog	Thailand	2010
	CPV-2c	CU24	MH711894	Dog	Thailand	2016
	CPV-2c	CU21	MH711902	Cat	Thailand	2016
	CPV-2c	TRC-B90/TH/202	MW589468	Dog	Thailand	2020
	CPV-2c	CPV/dog/HCM/2/2013	LC216904	Dog	Indonesia	2013
	CPV-2b	Coonamble/NewSouthWales/Canisfamiliarias/2016	MN259001	Dog	Australia	2016
	CPV-2b	Coonamble/NewSouthWales/Canisfamiliarias/2015	MN258995	Dog	Australia	2015
	CPV-2c	K01708-1	MK144544	Dog	South Korea	2017

CPV-2c	HN07	MT106228	Dog	Vietnam	2017
CPV-2c	CPV-SH1516	MG013488	Dog	China	2017
CPV-2c	Canine/China/14/2017	MH476583	Dog	China	2017
CPV-2c	Canine/China/18/2017	MH476587	Dog	China	2017
CPV-2c	CPV-AHhf27	MT648203	Dog	China	2019
CPV-2c	JSYZ-69	MW017581	Dog	China	2019
CPV-2c	China-XA-1	MZ506743	Dog	China	2019
CPV-2c	CPV-SH2001	MW650830	Dog	China	2020
CPV-2c	CPV-SH2003	MW811189	Dog	China	2020
CPV-2c	ZJHN-136	MW017617	Dog	China	2020
CPV-2c	CPV_IZSSI_2743_17	MF510157	Dog	Italy	2017
CPV-2c	CPV609	MN451682	Dog	Nigeria	2018
CPV-2c	CPV605	MN451678	Dog	Nigeria	2018
CPV-2c	IZSSI_PA1464/19_idV4_TR_4A72	MT840293	Dog	Nigeria	2018
CPV-2c	IZSSI_PA1464/19_idYV7_TR_4A72	MT840294	Dog	Nigeria	2018
CPV-2c	157/2019	MW659469	Dog	Romania	2019
CPV-2c	158/2019	MW659470	Dog	Romania	2019
CPV-2c	159/2019	MW659471	Dog	Romania	2019
CPV-2c	160/2019	MW659472	Dog	Romania	2019
CPV-2c	161/2019	MW659473	Dog	Romania	2019
CPV-2c	162/2019	MW659474	Dog	Romania	2019
CPV-2c	163/2019	MW659475	Dog	Romania	2019
CPV-2c	164/2019	MW659476	Dog	Romania	2019
CPV-2c	165/2019	MW659477	Dog	Romania	2019
CPV-2c	166/2019	MW659478	Dog	Romania	2019
